# Single injection of small-molecule amyloid accelerator results in cell death of nigral dopamine neurons in mice

**DOI:** 10.1038/npjparkd.2015.24

**Published:** 2015-12-17

**Authors:** Maria Chermenina, Erik Chorell, Małgorzata Pokrzywa, Henrik Antti, Fredrik Almqvist, Ingrid Strömberg, Pernilla Wittung-Stafshede

**Affiliations:** 1 Department of Integrative Medical Biology, Umeå University, Umeå, Sweden; 2 Department of Chemistry, Umeå University, Umeå, Sweden; 3 Airoptic Sp. z o.o., ul. Rubież 46H, Poznań, Poland

## Abstract

The assembly process of α-synuclein toward amyloid fibers is linked to neurodegeneration in Parkinson’s disease. In the present study, we capitalized on the *in vitro* discovery of a small-molecule accelerator of α-synuclein amyloid formation and assessed its effects when injected in brains of normal mice. An accelerator and an inhibitor of α-synuclein amyloid formation, as well as vehicle only, were injected into the striatum of normal mice and followed by behavioral evaluation, immunohistochemistry, and metabolomics up to six months later. The effects of molecules injected into the substantia nigra of normal and α-synuclein knock-out mice were also analyzed. When accelerator or inhibitor was injected into the brain of normal mice no acute compound toxicity was found. However, 6 months after single striatal injection of accelerator, mice sensorimotor functions were impaired, whereas mice injected with inhibitor had no dysfunctions. Injection of accelerator (but not inhibitor or vehicle) into the substantia nigra revealed significant loss of tyrosine hydroxylase (TH)-positive neurons after 3 months. No loss of TH-positive neurons was found in α-synuclein knock-out mice injected with accelerator into the substantia nigra. Metabolic serum profiles from accelerator-injected normal mice matched those of newly diagnosed Parkinson’s disease patients, whereas the profiles from inhibitor-injected normal mice matched controls. Single inoculation of a small-molecule amyloid accelerator may be a new approach for studies of early events during dopamine neurodegeneration in mice.

## Introduction

Parkinson’s disease (PD) is the second most common neurological disorder and the most common movement disorder. It is characterized by widespread degeneration of subcortical structures of the brain, especially dopaminergic neurons in the substantia nigra. The assembly process of the intrinsically unstructured protein α-synuclein has been linked to the molecular basis of PD. α-Synuclein is a major component of the amyloid aggregates found in Lewy-body inclusions, which are pathological hallmarks of PD, and mutations in α-synuclein are related to familial PD cases.^1,2^ The exact function of α-synuclein is unknown, but it is suggested to be involved in synaptic vesicle release and trafficking, physiological regulation of enzymes and transporters, and participating in neuronal survival by controlling the neuronal apoptotic response^3^ and appears to be present in soluble and membrane-associated forms at presynaptic nerve terminals.^4–6^

α-Synuclein can assemble via oligomeric intermediates to amyloid fibrils and, finally, to inclusion bodies under pathological conditions.^7^ Although soluble α-synuclein oligomers have been proposed to be the most toxic species in PD-related neurodegeneration,^8,9^ recent work in animal models with preformed α-synculein fibrils have demonstrated that the amyloid fibrils themselves are toxic and can amplify *in vivo*, transmit to other cells, and cross the blood–brain barrier.^10–12^ Despite the lack of a mechanistic understanding of PD, many studies have focused on small synthetic or natural molecules that inhibit α-synuclein monomers to assemble into toxic oligomers and/or amyloid fibrils, or divert the α-synuclein assembly process toward nontoxic inert aggregates, as leads to counteract the disease.^13,14^ Inversely, the identification of small molecules that promote aggregation of α-synuclein into oligomers and amyloid fibers could be helpful as research tools for elucidation of early events during PD development in animals. Current animal models of PD are limited to studies of later events during disease progression as they either involve the use of toxic chemicals with non-synuclein targets that directly kill neurons or non-physiological overexpression of the α-synuclein polypeptide.^15–18^

**Figure 1:**
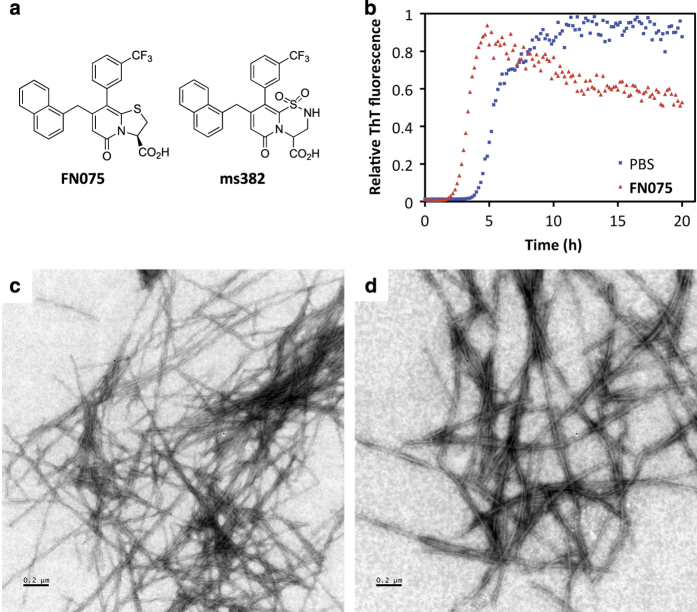
(**a**) Structures of the ring-fused 2-pyridones FN075 and ms382. (**b**) ThT fluorescence data for mouse α-synculein amyloid fiber formation in the absence (blue) and presence (red) of 1:1 molar ratio of FN075. (**c**) EM image of α-synculein fibers after an aggregation experiment. (**d**) EM image of α-synuclein fibers formed in the presence of FN075.

FN075 is a small synthetic molecule that promotes α-synuclein amyloid formation *in vitro* via rapid formation of soluble oligomers.^19^ Recently, small-angle X-ray scattering data demonstrated that the FN075-initiated oligomers were structurally very similar to α-synuclein oligomers formed without FN075 and, as an indication of toxicity, they readily caused leakage of lipid vesicles *in vitro.* ^19,20^ FN075 has a dihydro thiazolo ring-fused 2-pyridone central fragment designed to mimic a small C-terminal peptide with an extended β-sheet conformation (Figure 1a).^19,21,22^ Small chemical modification to the FN075 central fragment changes the properties so the molecule becomes an inhibitor of α-synuclein aggregation.^23^ Over the years, we have performed several *in vitro* characterizations of structure–function relationships for designed peptidomimetic 2-pyridone compounds on different amyloidogenic proteins, with an emphasis on α-synculein.^19,22–24^

In the present study, we tested whether injection of the amyloid accelerator FN075 into mice brains would promote neuronal damage and symptoms similar to early PD. We discovered that a single injection of FN075 into normal mice, months later, resulted in loss of dopoaminergic neurons, behavioral dysfunction, and metabolic serum profiles that paralleled those of newly diagnosed PD patients. None of these effects were found upon injection of a α-synuclein amyloid inhibitor molecule (Figure 1a), or when FN075 was injected into α-synuclein knock-out (*Snca* KO) mice.

## Results

### FN075 accelerates amyloid fiber formation *in vitro*

FN075 accelerates formation of amyloid fibers of human α-synuclein via rapid triggering of soluble α-synculein oligomers that then readily progress to fibers at aggregation-promoting conditions.^19,20^ To assure the same effects of FN075 on mouse α-synuclein, which has seven changes in its sequence compared with the human α-synuclein sequence, we expressed and purified recombinant mouse α-synuclein and tested the effect of FN075 on its amyloid fiber formation reaction using an *in vitro* aggregation assay based on Thioflavin T fluorescence. As expected, FN075 also accelerated amyloid fiber formation by mouse α-synuclein *in vitro* (Figure 1b). The resulting amyloid fibers formed in the presence of FN075 appear similar to those formed without FN075 according to electron microscopy (EM) data (Figures 1c and d).

### Brain targets for small-molecule injection

To determine where to inject small molecules, we measured α-synuclein protein levels in different brain regions of normal mice. The levels of α-synuclein were significantly higher in the striatum as compared with the cerebellum (*P*<0.0001; Bonferroni *post hoc* test). The age of the mice did not influence the α-synuclein levels (F_3,28_=0.162, *P*>0.05; two-way analysis of variance (ANOVA); Supplementary Figure S1). Because of the high levels of α-synuclein, the striatum was selected as primary target for injection of FN075, ms382, or vehicle. For comparison, molecules were injected into the substantia nigra of normal and α-synuclein-lacking mice to study direct effects on the nigral neurons and the role of α-synuclein.

### Sensorimotor dysfunction

**Figure 2:**
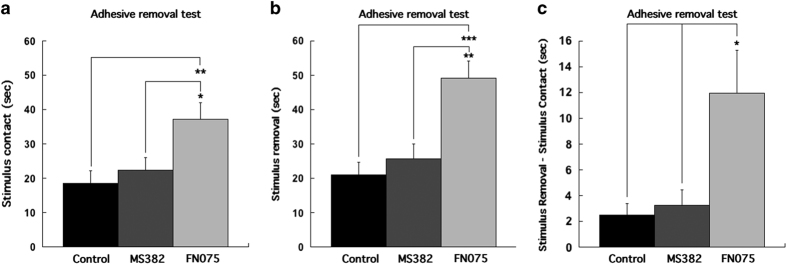
Data from behavioral adhesive removal test (**a**–**c**). The time to contact stimulus (**a**), the time to remove stimulus (**b**) and the time from stimulus contact to stimulus removal (**c**) were significantly longer in mice injected with the accelerator than the inhibitor and empty vehicle.

Early behavioral impairment can be detected with the adhesive removal test, which has high sensitivity to sensorimotor dysfunction,^25^ detecting effects not observed by the rotarod and open-field tests.^26^ The basis for the adhesive removal test is the fact that in response to adhesive stimulus on the nose, normal mice raise both forelimbs toward their snout and quickly remove the stimulus with both forepaws. The sticker was removed if the animal did not swipe it off within 60 s. The time to react to the stimulus contact was significantly different between animals given the different treatments (F_2,27_=5.917, *P*<0.01; one-way ANOVA). Animals given the accelerator were significantly slower (37.2±4.7 s) to react to the contact stimulus compared with control mice (18.5±3.7 s; *P*<0.01; Bonferroni *post hoc*) and mice injected with the inhibitor (22.4±3.7 s; *P*<0.05). Moreover, the time to remove the stimulus was significantly different (F_2,27_=11.896, *P*<0.001), where the time to remove the stimulus was significantly longer for mice injected with the accelerator (49.2±4.9 s) when compared with mice injected with the inhibitor (25.6±4.7 s; *P*<0.01) and vehicle controls (21.0±3.7 s; *P*<0.001, Bonferroni *post hoc* test). In addition, the calculated stimulus-removal minus stimulus-contact time was also significantly longer for animals injected with the accelerator (12.0±3.3 s) compared with inhibitor (3.2±1.2 s; *P*<0.05) and controls (2.5±0.9 s; *P*<0.05; one-way ANOVA: F_2,27_=6.163, *P*<0.01; Figures 2a–c). Movies of a typical FN075-treated mouse and a control mouse performing sticker tests are shown in Supplementary Information: Supplementary Movie S1 (FN075-treated) and Supplementary Movie S2 (control).

### Loss of dopamine-producing neurons

**Figure 3:**
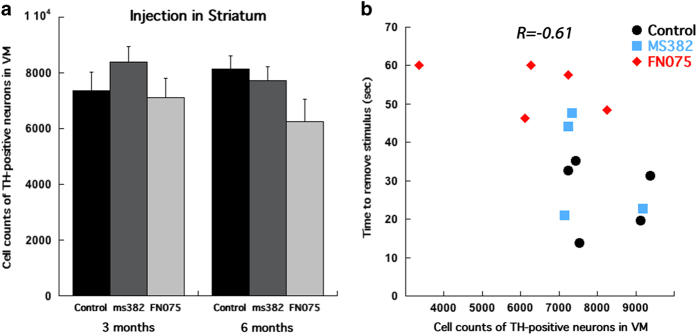
Cell counts of TH-positive neurons in the substantia nigra of mice injected with the accelerator, inhibitor, and vehicle into the striatum revealed (**a**) no significant difference between the treatments at 3 months (*n*=7 for vehicle, *n*=5 for inhibitor and *n*=9 for accelerator), but a clear decreasing trend at 6 months post-injection (*n*=5 for vehicle, *n*=4 for inhibitor and *n*=5 for accelerator). Scatter plot demonstrates a significant negative correlation (*R*=−0.61, *P*<0.05) between the time to remove the stimulus in adhesive removal test and counts of TH-positive neurons in the substantia nigra (**b**), (*n*=5 for vehicle, *n*=4 for inhibitor, and *n*=5 for accelerator). *P<0.05, **P<0.01, ***P<0.001.

Two time points, 3 and 6 months post small-molecule injection into the striatum were chosen to evaluate survival of tyrosine hydroxylase (TH) -positive (i.e., dopamine producing) neurons in the ipsilateral substantia nigra. The analysis revealed no significant difference in survival of TH-immunoreactive cells between the treatments at 3 months after the injection (F_2,19_=0.759; *P*>0.05; one-way ANOVA; Figure 3a). At 6 months, a moderate reduction of TH-positive neurons was found in mice injected with FN075 as compared with control and inhibitor-injected mice (F_2,11_=2.625; *P*>0.05; one-way ANOVA; Figure 3a). The number of TH-positive neurons and the adhesive removal test result exhibited a significant correlation such that mice that performed slower in the behavioral test also possessed fewer TH-positive neurons in the substantia nigra (linear correlation coefficient *R*=−0.61, *n*=14, *P*<0.05). Linear regression demonstrated a significant relationship (F(1,12)=7.232, *P*<0.05; Figure 3b).

**Figure 4:**
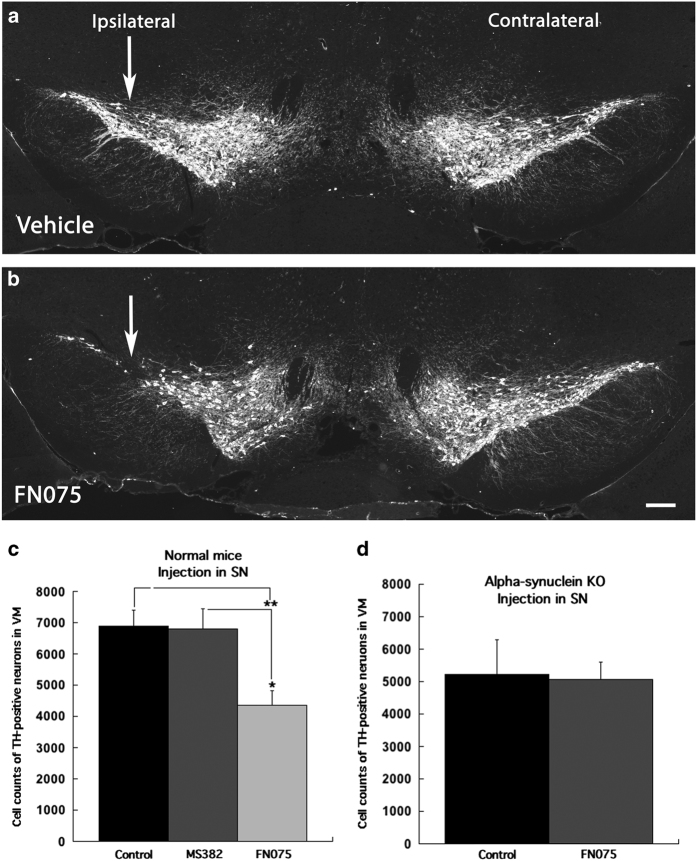
TH-immunohistochemistry of the substantia nigra of vehicle injection (**a**) and FN075 injection (**b**) into the substantia nigra. The depletion of TH-positive neurons is seen ipsilateral to the accelerator injection (left in **b**). Almost 50% reduction in counts of TH-positive neurons was found in mice injected with the accelerator compared with controls and animals treated with the inhibitor at 3 months after substantia nigra injection (*n*=6 for vehicle, *n*=4 for inhibitor, and *n*=6 for accelerator; **c**). In contrast to the significant reduction of TH-positive neurons in normal mice, no differences in the number of TH-positive neurons was found in *Snca* KO mice after injection of FN075 near substantia nigra at 3 months postinjection (*n*=6 for vehicle and *n*=6 for accelerator, **d**). *P<0.05; **P<0.01. Scale bars: (**a** and **b**) 100 μm.

The nigral cell death was more pronounced when FN075 was injected near the substantia nigra. Cell counts of TH-positive neurons in the substantia nigra, ipsilaterally to the injection, of such mice were significantly reduced (4,347±473 cells) already at 3 months post injection as compared with controls (6,889±509 cells*; P*<0.01) and to mice injected with inhibitor ms382 (6,800±639 cells; *P*<0.05; one way ANOVA: F_2,11_=7.893 *P*<0.01) (Figures 4a–c). To assure that the loss of TH-positive cells in the FN075-injected mice was due to neuronal cell death and not loss of TH expression, TH-immunoreactive sections were also processed for NeuN, as well as stained for cresyl violet (Supplementary Figure S2), which clearly visualized loss of neurons in the substantia nigra of FN075-injected mice.

### Neuronal damage requires α-synuclein

To test if the loss of TH-positive nigral neurons in normal mice injected with the accelerator depended on the presence of α-synuclein, cell counts of nigral neurons were also performed in *Snca* KO mice that lack α-synuclein, which had been treated with FN075 in the same way as the normal mice. The results revealed that the number of TH-positive neurons in the *Snca* KO mice that received injection of FN075 near the substantia nigra (5,060±531) was not different from the number of neurons counted in the *Snca* KO mice that received only vehicle (5,213±1078; *t*_10_=0.128; *P*>0.05; Independent Student’s *T*-test) at 3 months after the treatment (Figure 4d). This strongly implies that α-synuclein is required for FN075 to exert its effects in the normal mice, although one should be aware that there are many differences at the transcriptional level between these mice. As expected, the *Snca* KO mice did not show any significant differences in their adhesive removal performances due to FN075 treatment (Supplementary Figure S3).

Complementary support for the FN075 effects being mediated via α-synuclein comes from independent experiments in another *in vivo* model. Fruit flies overexpressing the human α-synuclein gene and fed with FN075 were found to have shorter median life span (i.e., FN075 mediates toxicity) and increased total levels of α-synuclein protein (FN075 promotes accumulation of degradation-resistant forms) as compared with vehicle-fed flies and flies fed with an inhibitor molecule (Supplementary Figures S4 and S5; Supplementary Materials). Although we did not test the effect of FN075 on normal flies, the lack of toxic effects by the inhibitor molecule in the overexpressing flies imply that the observed effects are not due to a general effect of this type of molecules.

### Serum metabolomics match PD patients

**Figure 5:**
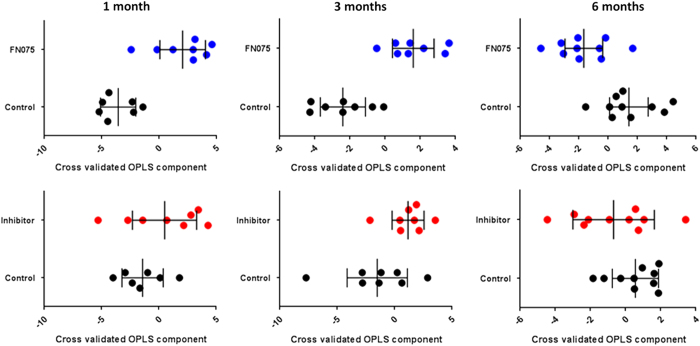
Data from metabolomics analysis. Upper row; Cross-validated OPLS-DA scores for pairwise OPLS-DA models between control mice (black dots) and FN075 injected mice (blue dots) (*P*_1month_=0.02, *P*_3months_=0.03, *P*_6months_=0.06; based on ANOVA of cross-validated models). Lower row; Cross-validated OPLS-DA scores for pairwise OPLS-DA models between; control mice (black dots) and inhibitor-injected mice (red dots) (*P*_1month_=0.23, *P*_3months_=0.17, *P*_6months_=0.71; based on ANOVA of cross validated models). Each sample class is described by the average and 95% confidence interval. In all plots each symbol represents one serum sample described by the whole-measured metabolite profile.

Serum metabolomics data on samples taken at 1, 3, and 6 months post-injection of molecules into the striatum of the mice were analyzed for low-molecular-weight compounds (metabolites). The serum samples report on effects on homeostasis of the whole body and the underlying mechanisms relating perturbations in the nigrostriatal dopamine system to serum metabolite levels are not known. Multivariate analysis (Orthogonal partial least squares discriminant analysis (OPLS-DA)) revealed serum metabolite profile changes indicating that while the FN075-treated mice samples show a significant change in relation to control, such effects are not statistically significant in the inhibitor-treated mice (Figure 5). FN075-treated mice showed increases in several amino acids at the early stages post-injection (arginine, ornithine, lysine, tryptophan, serine, tyrosine, valine, glutamine, phenylalanine, asparagine, isoleucine, alanine, and pyroglutamic acid) as well as decreases in C_15_–C_18_ saturated and unsaturated fatty acids (including pentadecanoic acid, hexadecanoic acid, and linoleic acid) as compared with the inhibitor-treated and control mice. Six months post injection, the metabolic profile of the FN075-treated mice (compared with the controls and inhibitor treated) now displayed decreased levels of amino acids as well as increased blood sugar, hippuric acid, and cholesterol contents.

Although mechanistic interpretations are difficult, the metabolite changes at the early stages following injection for the FN075-treated mice match metabolic profiles obtained in newly diagnosed Parkinson’s disease patients in terms of both identity of altered metabolites and direction of change.^27^ In contrast, analysis of serum from *Snca* KO mice demonstrated no significant difference in metabolic profile (*P*=0.136) when comparing samples from 3-month-old FN075-injected mice with vehicle-injected mice.

## Discussion

In addition to finding biomarkers and treatments for neurodegenerative diseases such as Alzheimer’s and Parkinson’s disease, it is of high importance to also develop new research tools that allow better characterization of the molecular steps involved in the disease progression. In the present study, we investigated the consequences on the nigrostriatal dopamine system of normal mice upon a single injection of a small peptidomimetic molecule, FN075. On the basis of the *in vitro* observations that FN075 promotes formation of α-synuclein oligomers and amyloid fibers,^19,23^ we speculated that mice injected with FN075 would develop PD-like symptoms. In agreement, we discovered that FN075-injected mice had impaired sensorimotor functions, a vast loss of TH-positive neurons, and metabolic serum profiles that matched those of early-diagnosed PD patients.^27^ Although the molecular mechanisms behind these effects are unclear, the observations of no nigral cell death and no change in metabolic profiles for *Snca* KO mice injected with FN075 clearly indicate that the FN075 effects found in normal mice are dependent on α-synuclein interactions. The fruit fly data are also in agreement with FN075 targeting α-synuclein as the total α-synuclein protein levels were increased, along with reduced life span, upon FN075 feeding of the flies.

Established mouse models of PD include the use of toxic chemicals, overexpression of wild type or mutated α-synuclein, or exogenously added α-synuclein formulas.^17,18^ The different models all have their strengths and limitations and one has to carefully correlate the phenotypic presentations (i.e., neuronal death, Lewy bodies,^18,28^ motor symptoms) of the model with the research questions to be asked. For example, α-synuclein transgenic mice have failed to demonstrate loss of dopaminergic neurons,^29–31^ whereas overexpression of α-synuclein via viruses resulted in robust neurodegeneration along with protein aggregation.^32,33^ Moreover, when human wild-type α-synuclein was overexpressed under different promoters, there was divergent results concerning the presence or not of α-synuclein inclusions.^18,34^ However, in mice overexpressing disease-causing mutants of α-synuclein,^35^ inclusions resembling Lewy bodies were detected.^36^ Our approach differs from available animal models in that it involves a single injection of a small molecule into normal mice with the purpose of affecting endogenous α-synuclein in the brains of these mice. In some analogy to our approach, recent studies have elegantly demonstrated that injections of preformed α-synculein fibrils in animal brains cause PD pathology, and it was found that injected fibrils could transmit their toxic structural properties to endogenous α-synuclein polypeptides.^10–12^

Whereas we found neuronal cell death upon FN075-injection, none of the injected mice developed detectable amounts of α-synuclein inclusions or Lewy bodies as probed by antibodies for phosphorylated α-synuclein and ubiquitin (data not shown). This suggests that the FN075-injected mice are in early stages of the disease where no Lewy bodies have accumulated. In accord, deficits in dopaminergic neuron activity have been reported prior to neuronal loss.^28^ Although we cannot exclude other explanations, the molecular mechanism may involve FN075-triggered α-synuclein oligomers as the toxic species, but at concentrations too low to be detectable by conventional methods. In support, it was recently demonstrated that α-synuclein oligomers were present in neurons of PD patients prior to Lewy-body formation although a more sensitive detection method had to be developed to capture these oligomers.^37^ The similarity of the metabolomics data with that of PD patients strongly implies that FN075-injection promotes PD-like symptoms; this also gives indirect support for the involvement of α-synuclein. Notably, at 6 months post injection of FN075, the serum levels of several blood sugars, cholesterol and hippuric acid had increased. These compounds are all reported as potential markers of diabetes^38–40^ and, although more experiments are needed, may support the proposed link between type 2 diabetes and PD.^41,42^

## Conclusions

We here evaluated the nigrostriatal dopamine system in normal mice upon single injection of a small-molecule accelerator of α-synuclein amyloid formation *in vitro*. Complementary experiments supported that the accelerator effects *in vivo* are dependent on the presence of α-synuclein. Taken together, our observations suggest that accelerator-injected mice adopt symptoms equaling early stages of PD (such as reduced sensorimotor function, loss of dopamine neurons, and altered metabolite profiles). We propose that this strategy constitutes a new approach for molecular studies of early events in neurodegeneration related to PD.

## Materials and methods

### *In vitro* methods

The mouse α-synuclein construct was ordered from GenScript (NJ, USA), expressed, and delivered in a pET-3a vector using the restriction sites NdeI-*BamH*I. The construct carries the gene for mouse α-synuclein co-expressed with the 434 repressor protein which also contains an N-terminal His-tag and a caspase 7 cleavage site. Plasmid was transformed into BL21 (DE3) competent cells and grown at 37 °C in 5× LB medium supplemented with 100 mg/ml carbenicillin and grown until OD_600_~0.6. Protein expression was induced with 0.5 mmol/l isopropyl b-D-1-thiogalactopyranoside, and the cells were further grown overnight. The cells were centrifuged for 30 min at 5000 rpm, and the pellet resuspended in 8 mol/l urea, 20 mmol/l Tris, 20 mmol/l imidazole, pH 8.0, and sonicated on ice, followed by centrifugation at 20,000 r.p.m. for 30 min. The supernatant was filtered and loaded on an affinity column (Ni Sepharose 6 Fast Flow, GE Healthcare, Uppsala, Sweden), equilibrated with 20 mmol/l Tris, 50 mmol/l NaCl, 20 mmol/l imidazole, 5% glycerol, pH 7.5, and eluted with the same buffer, but contained 250 mmol/l imidazole. For removal of the His-tag as well as co-expressed protein, peptidase caspase 7 was added in a ratio of 1:100 (w/w), together with 20 mmol/l 2-mercaptoethanol and was put to incubation over night at 4 °C. Cleavage efficiency was verified with SDS-polyacrylamide gel electrophoresis, and selected fractions were diluted 1:1 (v/v) with Milli-Q water (Molsheim, France). The sample was then loaded on a anion-exchange column (HiTrap Q FF, GE Healthcare) equilibrated with 20 mmol/l Tris pH 8.0, and eluted with a linear NaCl gradient of 20 mmol/l Tris, 1 mol/l NaCl, pH 8.0. Finally, α-synuclein was run through a gel filtration column (HiLoad 16/60 Superdex 75, GE Healthcare), equilibrated with 50 mmol/l ammonium carbonate. The α-synuclein concentration was determined using the absorption at 280 nm. α-synuclein amyloid formation assays were conducted at 37 °C with constant agitation with a 2-mm glass bead in each well (70 μmol/l synuclein, 20 μmol/l ThT). Graphs are representative of at least three independent experiments. Values are reported as normalized ThT fluorescence. For transmission electron microscopy, 15-μl samples from the fibrillation studies (diluted 1:10 with Milli-Q water) were applied to Formvar coated 200 mesh size copper grids. Incubated for 3 min and stained with 2% sodium silicon tungstate for 15 s before being allowed to dry. The grids were examined with a Jeol (Tokyo, Japan) model 1230 electron microscope operated at 80 kV. Images were obtained using a Gatan MultiScan 600 W camera (Abingdon, UK).

### Animals

C57Bl/6 (Charles River, Sulzfeld, Germany) and *Snca* KO (strain name: B6;129X1-*Snca* ^tm1Rosl^/J, The Jackson Laboratory, Maine, USA) female mice were used in this study. The mice were 8-week old when the study was initiated. They were housed in groups of five per cage with free access to food and water, and kept in 12- h light/dark cycle. The Northern Swedish Ethics Committee for animal experiment, in compliance with international guidelines for the protection of animals used for scientific purposes of the European Parliament and of the Council Directive (2010/63/EU), had approved all animal procedures (A 69-12 and A 24-15).

### Compounds to affect α-synuclein aggregation

The 2-pyridones FN075 and ms382 (and ms400, another inhibitor used in fly studies, see Supplementary Information) were synthesized as previously described.^21,23,43^ FN075 is an accelerator while ms382 and ms400 are inhibitors of α-synuclein aggregation.^19,23^ Earlier work has shown that these compounds are monomeric in solution at the concentration used here.^23,24^

### Intracranial injection

The molecules were injected unilaterally either into the striatum or the substantia nigra. Mice, subjected to the striatal injection, were randomized into 3 subgroups: the first group received 2 μl 1 mmol/l FN075 in 10% dimethyl sulfoxide (DMSO) diluted in 0.9% NaCl (the accelerator, *n*=14), animals in the second group received 2 μl 1 mmol/l ms382 in 10% DMSO diluted in 0.9% NaCl (the inhibitor, *n*=9), and the third group received 2 μl of 10% DMSO in 0.9% NaCl (controls, *n*=12). To perform nigral injections, animals were divided using the same strategy (*n*=6 for vehicle, *n*=4 for inhibito, and *n*=6 for accelerator). *Snca* KO mice were randomized into two groups (*n*=6 for vehicle and *n*=6 for accelerator) for injection into the substantia nigra. Prior to injection, the mice were anesthetized with isofluran (Baxter Medical AB, Kista, Sweden) and placed in a stereotaxic frame. A hole was drilled in the skull overlying the striatum or the substantia nigra. The coordinates for unilateral injection were: anterior-posterior (AP) +0.12 mm, mediolateral (ML) −0.17 mm, dorsoventral (DV) from the dura mater −0.30 mm and AP −0.24 mm, ML −0.14 mm, DV −0.45 mm for the striatum and substantia nigra, respectively. The injections were performed with a 10 μl syringe (701N, 26s/2"/3; Hamilton Company, Nevada, USA) at the rate of 1 μl/2 min. The needle was withdrawn after 2 min in order to avoid reflux.

### Behavioral analysis

Sensorimotor functions in mice injected into the striatum 6 months post-injection or *Snca* KO mice injected into substantia nigra 3 mounts post-injection were tested using adhesive removal test.^25^ An adhesive-backed stimulus in form of a quarter of a circle, 10 mm in diameter, was placed on the snout of the mouse. The time to sense and the time to remove the sticker were measured with the time limit of 60 s and removal-contact time was calculated. The time to sense (contact) shows sensory deficits, whereas the time to remove the stimulus indicates motor impairment.^44^ Animal’s home cage was used for the tests and all cage mates were moved temporarily to another cage. Each animal received three trials at three different days. The average time measured for these three trials were used to calculate the results.

### Tissue preparation and immunohistochemistry

Transcardial perfusion using Ca^2+^-free Tyrode solution followed by 4% paraformaldehyde in 0.1 mol/l phosphate buffer (pH 7.4) was performed under deep sodium pentobarbital anesthesia, and thereafter the mouse brains were dissected at 3 and 6 months after the intracranial injection of the small molecules. The brains were postfixed in 4% paraformaldehyde overnight and rinsed in 10% sucrose in 0.1 mol/l phosphate buffer with 0.01% sodium azide before sectioning. The brains were frozen with gaseous CO_2_ before being sectioned at 40 μm for stereology through the entire substantia nigra, and 14 μm thick sections were collected over both the striatal and substantia nigral areas for regular immunohistochemistry. The sections were rinsed in phosphate buffered saline (PBS; 0.1 M, pH 7.4) for 15 min before the primary antibodies were applied. The primary antibodies used were the dopamine cell marker tyrosine hydroxylase, raised in mouse against rat tyrosine hydroxylase (TH; 1:1,500; Cat # 22941; Immunostar Inc; Hudson, WI, USA), anti-alpha Synuclein (phospho S129) raised in rabbit monoclonal antibody [EP1536Y] (1:200; Cat # ab51253; Abcam, Cambridge, UK), anti-ubiquitin raised in rabbit polyclonal antibody (1:300; Cat # Z0458; Dako Cytomation, Glostrup, Denmark), anti-NeuN raised in rabbit polyclonal antibody (1:500; Cat # ab104225; Abcam, Cambrige, UK). The sections were incubated with primary antibodies diluted in 0.1 mol/l PBS with 0.3% Triton-X-100 for 48 h at 4 °C. After primary antibody incubations, the sections were rinsed and 5% goat serum in 0.1 mol/l PBS was applied to avoid unspecific binding. Alexa Fluor594 (1:500, Invitrogen, Molecular probes; Eugene, OR, USA) goat anti-mouse IgG (Cat # A11029), goat anti-rabbit IgG and Alexa Fluor488 goat anti-mouse IgG, or goat anti-rabbit IgG were used as secondary antibody. Finally, the sections were rinsed in PBS and mounted in 90% glycerol in PBS. Some sections were stained with cresyl violet to verify that neuronal death paralleled loss of TH phenotype in the nigral cells.

### Stereology

To estimate the total number of TH-positive cells in the substantia nigra ipsilateral to the injection, Optical Fractionator stereological method was used.^45^ Sections (40 μm) were collected at an interval of 1:6 over the entire substantia nigra and processed for TH-immunohistochemistry. The total number of TH-positive neurons was counted using Stereo Investigator software (StereoInvestigator 7, version 4.00; MicroBrightField, Inc. Vermont, USA). The substantia nigra was traced using an Olympus BX61 Microscope under low magnification using a ×10 objective. TH-positive neurons were then counted under a 60X objective with the following stereological parameters: grid size 180×180 μm; counting frame size 100.5×75.67 μm; dissector height 20 μm. The values of the coefficient of error (CE)<0.10 were accepted.

### Western blot

Cerebellum, striatum, and the ventral mesencephalon including the substantia nigra from C57Bl/6 mice of 6 and 22 months of age were used to quantify the amount of α-synuclein (*n*=6 for cerebellum, *n*=12 for striatum and *n*=12 for ventral mesencephalon). The mice were anesthetized with 4% isofluran (Baxter Medical AB, Sweden) and neck dislocated. The brains were dissected into the different brain regions and frozen in CO_2_. The brain tissues were sonicated (Sonifier B-12, Branson sonic power company, Danbury, CT, USA) after dilution with the lysis buffer (137 mmol/l NaCl, 2.7 mmol/l KCl, 8.1 mmol/l Na_2_HPO_4_, 1.5 mmol/l KH_2_PO_4_, pH=7.2–7.4, 1% IGEPAL CA-630, 10% glycerol, 1% protease inhibitor). The supernatant and pellet were separated by centrifugation in 10,000 r.p.m. for 15 min at 4 °C. α-synuclein was found in the supernatant. The BCA Protein Assay Kit (Pierce, Rockford, IL, USA) was used to quantify the protein level in the brain samples. An amount of 8 μg protein from each sample was mixed 1:1 with loading buffer (5% SDS, 10% 2-mercaptoehtanol, 20% glycerol, 0.005% bromophenol blue, 0.125 mol/l Tris HCl) and boiled 5 min at 95 °C to create a lysate to load on 12% SDS polyacrylamide gels. To determine the molecular weights of the proteins, Precision Plus Protein Standards Dual Color (Bio-Rad Laboratories AB, Solna, Sweden) was used. Transfer of proteins to a nitrocellulose membranes (Hybond ECL, Amersham Biosciences, Uppsala, Sweden) was performed using the Trans-Blot Semidry transfer (Bio-Rad Laboratories AB), followed by membrane blocking by 5% milk in tris buffer saline-tween (TBS-T) for 1 h at room temperature on a shaking table. The membranes were incubated with mouse anti-actin monoclonal antibody (1:10,000; Cat # MAB1501R; Chemicon International, Temecula, CA, USA) and rabbit anti-mouse α-synuclein (1:1,000; Cat # 2628; Cell Signaling, Danvers, MA, USA) overnight at 4 °C. Washing steps 3×5 min with TBS-T solution of the membranes were performed before the membranes were incubated with the secondary antibodies Immuno Pure IgG goat anti-mouse antibody (1:70,000, Cat # 31430; Pierce), polyclonal goat anti-rabbit immunoglobulins/HRP (1:10,000, Cat # P0448; Dako Cytomation) for 1 h. The membranes were rinsed in TBS-T 3×5 min and exposed to ECL plus western blotting detection reagents for 1 min and were then developed (developer and fixer, 1:5, Kodak, Sigma Aldrich onto Hyperfilm ECL (GE Healthcare Life Sciences, Uppsala, Sweden). Quantification of band signal intensity was performed using NIH ImageJ 1.45 s program (Softonic International SA, Barcelona, Spain).

### Evaluation and statistics

Throughout the experiments, all animals, slices and samples were blind-coded when analyzed. One-way ANOVA followed by Bonferroni *post hoc* test was used to estimate differences in behavior patterns and cell counts of TH-positive neurons in the substantia nigra. Two-way ANOVA was performed to find differences between treatments over time. The differences in level of α-synuclein protein in different brain tissue were calculated by two-way ANOVA with brain regions and time as factors followed by Bonferroni *post hoc* test. One-way ANOVA followed by Bonferroni *post hoc* test and Student’s *T*-test was used to reveal difference in cell counts in substantia nigra injection. All statistical tests were two-sided. A Person's product-moment correlation and linear regression were run to determine the relationship between the remaining number of TH-positive neurons and the adhesive removal test result. The null hypothesis was rejected at the 0.05 level. The results are expressed as mean value±s.e.m. SPSS 20 statistical software (IBM Corporation, NY, USA) was used to perform all statistical tests.

### Metabolomic analysis

The serum samples were extracted and derivatized according to the serum protocol for metabolomics available at Umeå Plant Science Centre (UPSC).^46^ Frozen 100-μl aliquots of serum, in Eppendorf tubes (Sarstedt Ref: 72.690), were first thawed in room temperature and then put on ice. Metabolite extraction was performed by addition of 900 μl methanol/water extraction mix (90:10 v/v) (including 11 isotopically labelled internal standards (7 ng/μl)) followed by rigorous shaking at 30 Hz for 2 min in a bead mill (MM 400, Retsch GmbH, Haan, Germany) and storage on ice for 120 min before centrifugation at 14,000 r.p.m. for 10 min at 4 °C (Centrifuge 5417 R, Eppendorf, Hamburg, Germany). Volume of 200 μl of the supernatants were transferred to GC vials and evaporated to dryness in a speedvac (miVac, Quattro concentrator, Barnstead Genevac, Ipswitch, UK). After evaporation the samples were stored at −80 °C until derivatization. Prior to derivatization the extracted serum samples were run for 5–10 min in the speedvac to remove possible condense. Methoxyamination, with addition of 30 μl methoxyamine in pyridine (15 μg/μl), 10 min of shaking and 60 min heating at 70 °C, was carried out with a 16 h reaction time (in room temperature). Trimethylsilylation, with addition of 30 μl MSFTA (*N*-methyl-*N*-trimethylsilyl-trifluoroacetamide)+1% Trimethylchlorosilane, was carried out for 1 h (in room temperature). Finally, 30 μl heptane including methyl stearate (15 ng/μl) was added as injection standard.

### GC(GC)/TOF-MS analysis

The extracted and derivatized serum samples were analysed either by GC/ or GCGC/TOFMS. For GC/TOFMS was 1 μl sample was injected splitless using an Agilent 7683 series autosampler (Agilent, Atlanta, GA, USA) into an Agilent 6980 GC with a 10 m×0.18 mm i.d. fused-silica capillary column chemically bonded with 0.18 μm DB5-MS stationary phase (J&W Scientific, Folsom, CA, USA). The injector temperature was set at 270 °C. Helium was used as carrier gas at (1 ml/min). Purge time was set to 60 s at a purge flow rate of 20 ml/min and an equilibration time of 1 min for every analysis. Initially, the column temperature was kept at 70 °C for 2 min and then increased to 320 °C at 30 °C/min for 2 min. The transfer line temperature was set at 250 °C and the ion source temperature at 200 °C. Ions were generated by a 70-eV electron beam at a current of 2.0 mA. Masses were acquired from m/z 50 to 800 at a rate of 30 spectra/s, and the acceleration voltage was turned on after a solvent delay of 165 s. For GCGC/TOFMS split less injection of 1 μl sample was performed using an Agilent 7683B auto sampler (injection temperature of 270 °C) into an a Pegasus 4D (Leco, St Joseph, MI, USA) with an Agilent 6890 gas chromatograph (Agilent Technologies, Palo Alto, GA, USA), a secondary GC oven and a quad-jet thermal modulator. Purge time was set to 60 s with a rate of 20 ml/min and helium was used as carrier gas (1 ml/min). The temperature program had an initial temperature of 60 °C for 2 min, followed by a increase of 4 °C/min up to 300 °C where the temperature was held constant for 2 min. The secondary oven used the same temperature program but with a +15°C offset. The modulation time was 5 s with a hot pulse time of 0.8 s and a 1.7 s cooling time between the stages. The MS transfer line temperature was 300 °C and the ion source 250 °C. 70 eV electron beams were used for the ionization and masses were recorded from 50 to 550 m/z at a rate of 100 spectra/s, with detector voltage set at 1780 V. All serum samples were run in a randomized order. Raw data was exported to Matlab (version 14B) and processed using multivariate curve resolution by means in-house developed scripts.^47^ Metabolites were identified by comparisons of detected spectral profiles and retention indices with available spectral libraries.

### Multivariate data analysis

Processed metabolomics data were subjected to further multivariate data analysis. OPLS-DA was performed to model the systematic variation in the metabolomics data related and orthogonal to pre-defined sample classes among the mouse serum samples, here considering, control versus FN075 treated as well as control versus inhibitor (ms382) at the three different time points. Furthermore, an OPLS-DA model was calculated to investigate the difference in metabolite profiles between FN075- and vehicle-injected *Snca* KO mice 3 months post injection following correction for run order bias. Cross validation was used to determine the predictive ability of the models, and ANOVA based on the cross-validated OPLS-DA models was employed to calculate *P*-values for the differences between the pre-defined sample classes in the respective models. The OPLS-DA model loadings were used to highlight significant metabolites. Results were displayed as cross-validated predictive scores for pairwise OPLS-DA models between control and FN075 injected mice as well as between control and inhibitor injected mice, respectively. Cross-validated predictive scores provide a visualization of the model robustness and thus of the validity of the detected metabolite patterns.
