# Supplementary material for: Single injection of small-molecule amyloid accelerator results in cell death of nigral dopamine neurons in mice
Source: NPJ Parkinsons Dis. 2015 Dec 17;1:15024–. doi: 10.1038/npjparkd.2015.24 (PMC5516562; doi:10.1038/npjparkd.2015.24)
Supplement: Supplementary Information [file npjparkd201524-s1.pdf]

Supplemental Information

Single injection of small-molecule amyloid accelerator results in cell death of nigral dopamine neurons in mice

by

Maria Chermenina, Erik Chorell, Małgorzata Pokrzywa, Henrik Antti, Fredrik Almqvist, Ingrid Strömberg, and Pernilla Wittung-Stafshede

Content

Figure S1

Figure S2

Figure S3

Figure S4

Figure S5

Supplemental Materials section (fly experiments)

Figure S1. Western blot was used to measure the levels of α-synuclein (**A, B**) in the cerebellum, VM, and the striatum in 6 and 22 month-old mice. The levels of α-synuclein were significantly higher in the striatum compared to the cerebellum at 6 and 22 months (**B**).

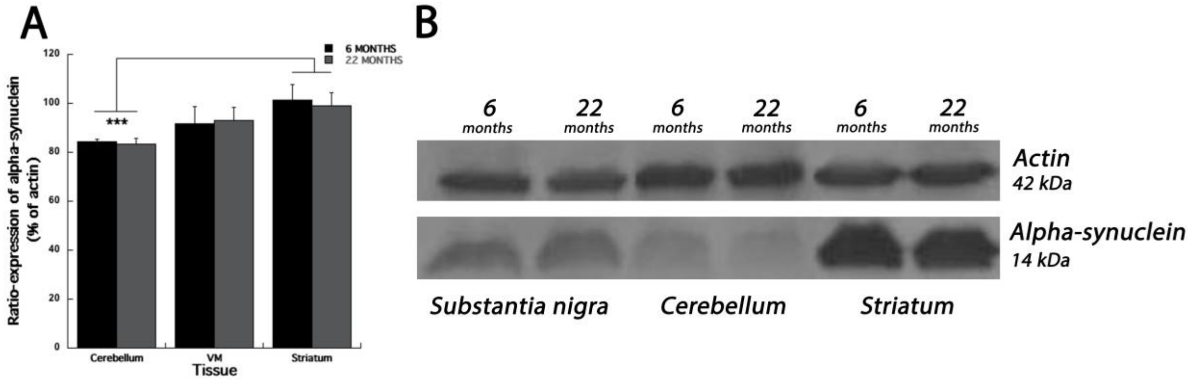

Figure S2. TH- (**A, C, E, H**) and NeuN- (**B, D, F, I**) immunohistochemistry and cresyl violet (**K, L, M, N**) in vehicle- (**A, B, E, F, G, K, M**) and FN075- (**C, D, H, I, J, L, N**) treated mice injected into the substantia nigra ipsilateral to the injection. TH-immunohistochemistry reveals that there is a loss of TH-positive neurons in animals injected with FN075 (**C**) compared to vehicle-injected controls (**A**). This loss was also true for NeuN-positive neurons (**B, D, F, I**), as well as in sections stained for cresyl violet. Arrows in **M** and **N** marks the substantia nigra. Scale bars for immunohistochemistry pictures A-D = 200 µm, E-J = 50 µm, K, L = 100 µm, M, N = 100 µm.

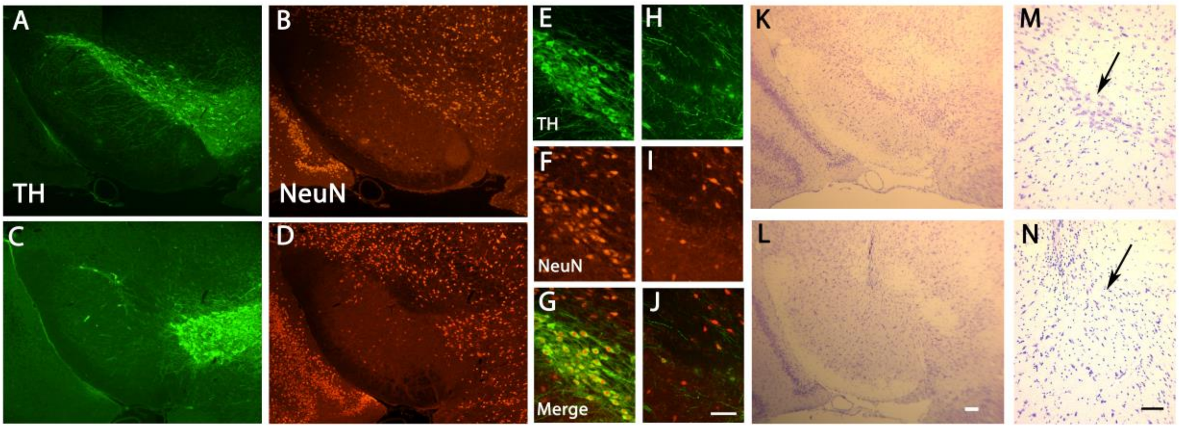

Figure S3. Data from adhesive removal test in *Snca* KO mice injected into substantia nigra 3 months post-injection. No significant difference was shown between the mice injected with FN075 and vehicle injected mice.

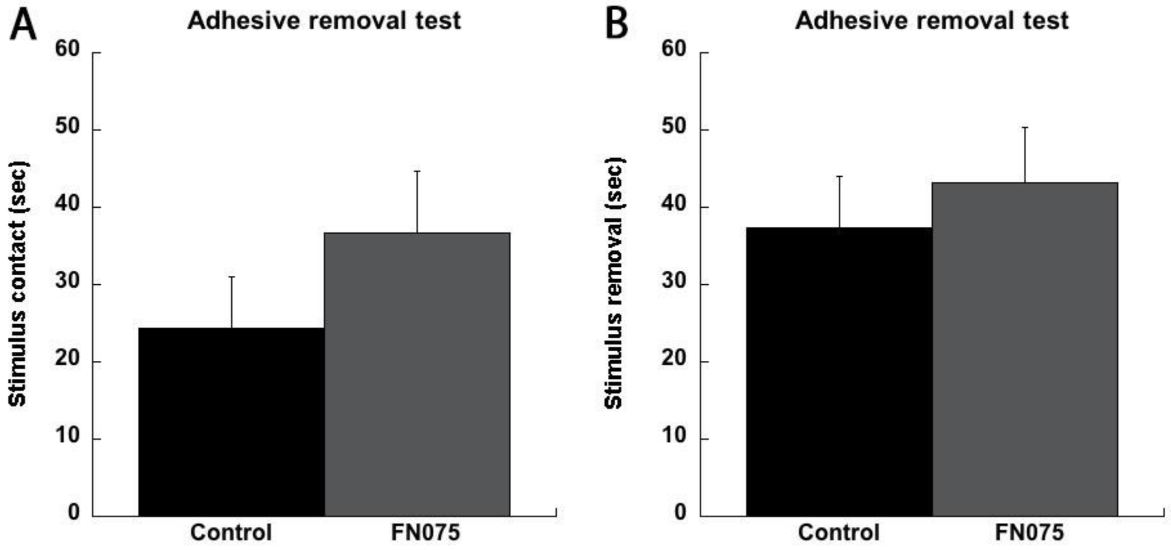

Fig**ure S4. Median lifetimes of flies expressing wild-type human α-synuclein (ASYN WT) fed with small molecules**. Flies expressing human wild-type α-synuclein in the CNS were fed with either FN075 or an inhibitor (abbreviated INH) at 50 µM and 100 µM concentrations in the food. Bars illustrate median values representing the number of days (given in insets) at which 50 % of flies had died in each small-molecule treatment set. ASYN WT flies fed with only vehicle had a median lifetime of 27 days which was shortened to 20 days for FN075-fed flies. Significance for Kaplan-Meier survival curves was estimated with the log rank statistical analysis, *p*-values $p < 0.001$ FN075 at 50 µM vs. vehicle-fed flies; $p = 0.061$ for INH at 100 µM vs. vehicle-fed flies).

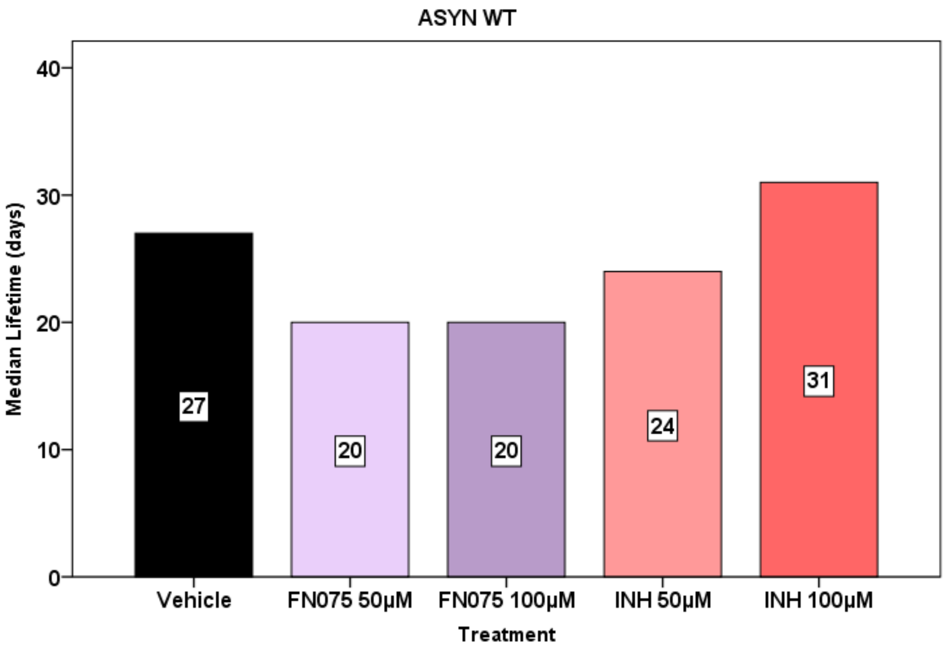

Figure S5. Quantification of total α-synuclein levels in protein extracts from heads of ASYN WT expressing flies fed with small-molecule compounds. Bars represent percent change in α-synuclein amounts (mean value ± S.E.M) in flies fed either FN075 or inhibitor (INH) vs. vehicle-fed flies. Flies were fed compounds at 50 µM and 100 µM concentrations and analyzed at days 10 and 20 of treatment for quantification α-synuclein amounts. Significant changes in α-synuclein levels were detected at day 20 for each treatment vs. vehicle-treated flies (GLM ANOVA with LSD post hoc test, $p = 0.027$ for FN075; $p = 0.003$ for INH 100 µM; $p = 0.035$ for INH 50 µM). Whereas FN075 fed flies had gradually increased α-synuclein levels at days 10 and 20 as compared to the control, inhibitor-fed flies had decreased α-synuclein levels at both time points.

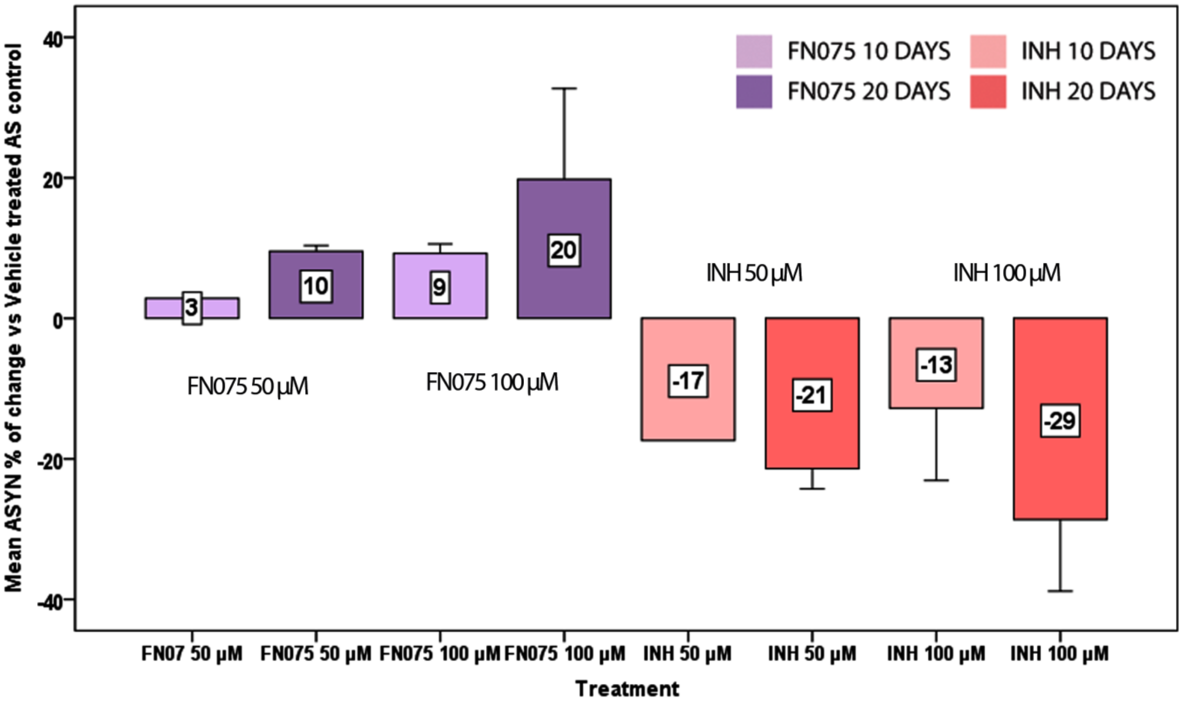

Supplementary Materials Section (fly experiments)

Drosophila stocks

Expression of α-synuclein (Bloomington stock #8146; *w[*]; P{w[+mC]=UAS-Hsap\SNCA.F}5B*) was obtained under control from pan-neuronal *n-syb-Gal4* driver line (kind gift of Dr Julie Simpson; Howard Hughes Medical Institute, MD).

Fly rearing and molecule feeding assay

Flies were kept at 60 % humidity at 20 °C under a 12:12 h light:dark cycle until eclosion and at 29 °C post eclosion. The crossings were reared in bottles containing standard *Drosophila* food (corn meal, corn syrup solids, yeast, water and agar). Newly emerged flies were transferred into 5 ml vials (10 flies per vial) containing low-melt fly food and the target compounds (accelerator FN075 and inhibitor MS400 (MS400 and MS382, inhibitor used in the mice, are structurally similar compounds that are both strong inhibitors of α-synuclein amyloid formation in vitro) according to formula developed by (Markstein, Dettorre et al. 2014) for mixing drugs in low volumes. Briefly, the food was prepared with distilled water containing 2 % (wt/vol) autoclaved yeast, 7 % (vol/vol) corn syrup liquids, and 1.5 % (wt/vol) agarose (composed of 1 part standard agarose to 11 parts low-melt agarose). The food was mixed as a liquid with the compounds at 37 °C. The two compounds were pre-dissolved in 95 % ethanol and mixed into the low-melt fly food at 50 or 100 μM final concentrations. The resulting food-compound mixtures solidified at 30 °C into soft fly-eatable gels. Every 2-3 days the flies were transferred to fresh vials and the number of dead flies was recorded throughout the lifespan of all flies. Graphs and statistical comparison were generated with IBM SPSS 20 Statistics (IBM Corporation, Armonk, NY).

Protein extraction and quantification of α-synuclein levels

Protein extracts were prepared according to a protocol modified from (Auluck, Meulener et al. 2005). Twenty fly heads from flies fed with compounds for 10 or 20 days were homogenized in 70 µl of extraction buffer (20 mM Tris pH 7.6, 50 mM NaCl, 1% Triton X-100, Protease inhibitor (Amresco LLC, OH)), vortexed gently and incubated on ice for 30 min. After centrifugation for 60 min at 15 x 1000 g in 4°C, supernatants were collected and mixed with 4x LDS Sample Buffer and DTT containing (10x) Sample Reducing Agent (Life Technologies, Carlsbad, CA). The remaining pellets were re-suspended in SDS extraction buffer (50 mM Tris pH 7.6, 5 mM EDTA, 4 % SDS), vortexed and boiled for 10 minutes. Supernatants were collected after centrifugation 10 min at 15 x 1000 g and mixed with 4x LDS sample buffer and DTT as described above. Both fractions were sonicated and boiled for 20 min before electrophoresis. For each extract a volume corresponding to 1,5 fly heads was resolved on NuPAGE® Novex® 4-12% Bis-Tris Protein Gels in MES SDS running buffer and electro-blotted onto Nitrocellulose membrane using iBlot2 gel transfer device (Life Technologies). All steps were performed according to the manufacturer. The primary antibodies used were mouse monoclonal against α-tubulin (1:5000, clone B-5-1-2, Life technologies) and rabbit polyclonal against human α-synuclein 1:1000 (AlexoTech AB). Detection was performed with the Western Breeze Chromogenic kit anti-mouse or anti-rabbit, respectively. α-synuclein levels were quantified by Gel-Doc XR+ Imager and Image Lab 5.2 software (Bio-Rad, Richmond, CA, USA). Folds of change in α-synuclein protein levels were normalized versus tubulin levels and presented as mean values ±S.E.M calculated as the percent of change (%) for drug-treated versus vehicle-treated flies from 2-3 independent drug feeding experiments. Graphs and statistical comparison were generated with IBM SPSS 20 Statistics (IBM Corporation, Armonk, NY).

References

*Auluck, P. K., M. C. Meulener, et al. (2005). "Mechanisms of Suppression of {alpha}-Synuclein Neurotoxicity by Geldanamycin in Drosophila." J Biol Chem **280**(4): 2873-2878.*

*Markstein, M., S. Dettorre, et al. (2014). "Systematic screen of chemotherapeutics in Drosophila stem cell tumors." Proc Natl Acad Sci U S A **111**(12): 4530-4535.*
